# Multi-trait analysis for genome-wide association study of five psychiatric disorders

**DOI:** 10.1038/s41398-020-00902-6

**Published:** 2020-06-30

**Authors:** Yulu Wu, Hongbao Cao, Ancha Baranova, Hailiang Huang, Sheng Li, Lei Cai, Shuquan Rao, Minhan Dai, Min Xie, Yikai Dou, Qinjian Hao, Ling Zhu, Xiangrong Zhang, Yin Yao, Mingqing Xu, Qiang Wang, Fuquan Zhang

**Affiliations:** 1 Mental Health Center and Psychiatric Laboratory, State Key Laboratory of Biotherapy, West China Hospital of Sichuan University, Chengdu, Sichuan China; 2 West China Brain Research Center, West China Hospital of Sichuan University, Chengdu, Sichuan China; 3 Department of Psychiatry, First Clinical Medical College/First Hospital of Shanxi Medical University, Taiyuan, China; 4 School of Systems Biology, George Mason University (GMU), Fairfax, VA USA; 5 Research Centre for Medical Genetics, Moscow, Russia; 6 Analytic and Translational Genetics Unit, Massachusetts General Hospital, Boston, MA USA; 7 Stanley Center for Psychiatric Research, Broad Institute of Harvard and MIT, Cambridge, MA USA; 8 Bio-X Institutes, Key Laboratory for the Genetics of Developmental and Neuropsychiatric Disorders (Ministry of Education), Shanghai Jiaotong University, 1954 Huashan Road, Xuhui, 200030 Shanghai, China; 9 School of Life Science and Engineering, Southwest Jiaotong University, Chengdu, China; 10 The Center of Gerontology and Geriatrics, West China Hospital of Sichuan University, Chengdu, Sichuan China; 11 Department of Geriatric Psychiatry, Nanjing Brain Hospital, Affiliated to Nanjing Medical University, Nanjing, China; 12 Collaborative Innovation Center of Genetics and Development, School of Life Sciences, Fudan University, Shanghai, China; 13 Shanghai Key Laboratory of Psychotic Disorders, Shanghai Mental Health Center, Shanghai Jiao Tong University School of Medicine, 600 South Wanping Road, Xuhui, 200030 Shanghai, China; 14 Department of Psychiatry, The Affiliated Brain Hospital of Nanjing Medical University, 264 Guangzhou Road, Nanjing, China

**Keywords:** Psychiatric disorders, Clinical genetics

## Abstract

We conducted a cross-trait meta-analysis of genome-wide association study on schizophrenia (SCZ) (*n* = 65,967), bipolar disorder (BD) (*n* = 41,653), autism spectrum disorder (ASD) (*n* = 46,350), attention deficit hyperactivity disorder (ADHD) (*n* = 55,374), and depression (DEP) (*n* = 688,809). After the meta-analysis, the number of genomic loci increased from 14 to 19 in ADHD, from 3 to 10 in ASD, from 45 to 57 in DEP, from 8 to 54 in BD, and from 64 to 87 in SCZ. We observed significant enrichment of overlapping genes among different disorders and identified a panel of cross-disorder genes. A total of seven genes were found being commonly associated with four out of five psychiatric conditions, namely *GABBR1*, *GLT8D1*, *HIST1H1B*, *HIST1H2BN*, *HIST1H4L*, *KCNB1*, and *DCC*. The SORCS3 gene was highlighted due to the fact that it was involved in all the five conditions of study. Analysis of correlations unveiled the existence of two clusters of related psychiatric conditions, SCZ and BD that were separate from the other three traits, and formed another group. Our results may provide a new insight for genetic basis of the five psychiatric disorders.

## Introduction

Schizophrenia (SCZ), bipolar disorder (BD), and major depressive disorder (MDD), are severe psychiatric disorders that commonly occur in late adolescence and early adulthood. Attention deficit hyperactivity disorder (ADHD) and autism spectrum disorder (ASD) are serious developmental disorders mainly influencing children. They are all leading causes of disability worldwide. A high proportion of susceptibility to these diseases can be explained by genetic factors, with an estimated heritability of 80%, 85%, 37%, 83%, and 74% for SCZ, BD, MDD, ASD, and ADHD, respectively^1–5^. Genome-wide association studies (GWASs) have revealed a few variations for a series of mental disorders. However, identification of contributing genetic loci has proved to be difficult, possibly due to the insufficient sample size, even if the assessed populations are increasing. Only a limited number of large-scale GWASs have been conducted for each trait. Although large-scale GWASs for ASD^6^ and ADHD^7^ have just been reported, the sample size is still relatively limited compared with other disorders such as SCZ and MMD.

It is well-known that the performance of standard GWAS concentrates on analyzing single nucleotide polymorphism (SNP) associate with a single trait, it may improve with an increase in the size of the cohort and in the degree of phenotypic similarity^8^. The multi-trait analysis of GWAS (MTAG)^9^ enables joint analysis of multiple traits, thereby boosting statistical power to detect genetic associations for each trait analyzed MTAG has significantly attracted scholars’ attention by a unique combination of four advantageous features compared with the other existing multi-trait analytic approaches^10–13^. It includes using GWAS summary statistics as an input, employing bivariate linkage disequilibrium (LD) score regression^14^ to compensate for an overlap of the cohorts described by different summary statistics, generating trait-specific effect estimate for each SNP, and taking relatively short computation time. The enhanced statistical power gained from combining data of related phenotypes has been previously reported^9,15^.

Moreover, several lines of evidence have shown that there existed phenotypic association amongst mental illnesses: (1) illusions or hallucinations can be a part of at least three different diagnoses—schizophrenia, bipolar disorder, and psychotic depression^16^; (2) it has been supported by family, twin and adoption studies that relatives of an affected proband were at higher risk of multiple disorders^17^ and a range of psychiatric disorders clustered together in families; (3) Schizoaffective patients possess both symptoms of SCZ and affective disorder^18^. In addition, varied degree of genetic correlations among the five disorders have been reported^19,20^.

On the other hand, in order to develop more optimized diagnostic criteria, it is highly essential to explore the correlations of heritability amongst different mental illnesses. For instance, previous studies on Psychiatric Genomics Consortium found that the overlap between heritability of SCZ and BD reached 68%, while the overlap between the heritability of the two illnesses and depression (DEP) was relatively lower^19^. It greatly supported the elimination of mood disorders in DSM-5 and the independence of bipolar disorder from DEP and related disorders. Given the potential phenotypic and genetic associations, we updated the analyzes of genetic correlations by using larger sample size and newer methodology in the current study. The present research will enrich the research domain criteria (RDoC) and assist to promote clinical and basic research on mental disorders.

Furthermore, since the above-mentioned five psychiatric disorders have genetic relationship with each other, they may share genetic loci and genes. However, genes that shared multiple disorders and the extent sharing common genes have largely remained unknown. Detecting common genes across major psychiatric traits may facilitate our understanding about possible common genetic basis of these traits.

To sum up, in this meta-analysis study, we performed a series of independent and joint analyzes of SCZ, BD, ASD, ADHD, and DEP. In addition to identification of novel variants and those identified by the GWASs, we also explored the genetic correlation and the overlap of risk genes among the five psychiatric disorders.

## Methods

### Samples

The summary statistics were obtained from GWAS of SCZ^8^, BD^8^, ASD^6^, ADHD^7^, and DEP^21^. Samples in the Psychiatric Genomics Consortium Major Depression (PGCMD) dataset included both MDD and DEP participants who were assessed by questionnaire (see Supplementary Figures and notes)^21^.

### Preprocessing of GWAS data

For non-rsID SNPs, we converted these SNP names using dbSNP147 (some non-rsID SNPs could not be converted). If multiple SNPs were mapped to an identical chromosomal position among different datasets, we dropped SNPs. SNPs with conflict alleles among different datasets were removed. *Z*-score was computed by log(OR)/SE. SNPs with minor allele frequency (MAF) differences of >0.2 among different datasets were removed. After variant filtering, the numbers of remained variants were 8,377,093, 8,956,949, 11,140,414, 9,087,710, and 8,028,859 for SCZ, BD, DEP, ASD, and ADHD, respectively.

### Multi-trait analysis of GWASs

MTAG^9^ a generalization of standard, inverse-variance-weighted meta-analysis, and takes GWAS summary statistics from an arbitrary number of traits. Herein, MTAG was applied to perform the meta-analysis of the five disorders. Bivariate LD score regression^14^ was used as part of an MTAG analysis to account for (possibly unknown) sample overlap between the GWAS results of different traits. In the result, MTAG outputs trait-specific effect estimates for each SNP and the resulting *P* value can be interpreted and used like those in single-trait GWAS. A total of 520,857 SNPs common among the five disorders were included in the MTAG analysis.

### Identification of significant loci by GWAS and their functional annotation

Functional Mapping and Annotation (FUMA)^22^ of GWAS Firstly, independent significant SNPs were identified on the basis of their *P* value, which were genome-wide significant (*P* ≤ 5.0 × 10^−8^) and independent from each other (*r*^2^ < 0.6) within a 1 mb window. Secondly, lead SNPs were identified as a subset of the independent significant SNPs that were in LD with each other at *r*^2^ < 0.1, again with a 1 mb window. Genomic risk loci were identified by merging lead SNPs if they were closer than 250 kb apart. Therefore, a genomic risk locus could contain multiple lead SNPs. To map LD, the 1000 Genomes Project was used^23^. ANNOVAR^24^ employed in FUMA was used to map SNPs to genes, and identify the function of the SNPs. Thirdly, other tools in FUMA were utilized to analyze the functional annotation, pathway, and tissue expression. All parameters were set as default.

FUMA included gene-based association analysis that was implemented by MAGMA 1.6 software^25^. Associated signals of SNPs within a gene were collapsed to derive a gene-based *P* value. Gene locations and boundaries were from the NCBI Build GRCh37 assembly. The European samples retrieved from the 1000 Genomes were used as reference dataset to account for LD between SNPs, and the potentially confounding effects of gene size and gene density were used as covariates. A Bonferroni correction was applied to control the multiple tests performed on the 18,770 genes available for analysis.

### Gene identification and gene-overlap analysis

Genome-wide significant genes were identified on the basis that one or more significant SNPs (*P* ≤ 5.0 × 10^−8^) were located within 2000 bp of a gene, or a gene with *P* value of gene-based association <2.66 × 10^−6^ (Bonferroni correction for 18,770 tests). Then significant genes were converted into official gene symbols using R package limma^26^. Genes that could not be mapped to an official gene symbols were removed.

In order to explore the locations of significant genes in chromosomal ideograms, we created chromosomal ideograms of significant genes, including SNP-based and MAGMA-based genes for GWAS and MTAG results using **PhenoGram**^27^.

Gene-overlap analysis among the five gene sets of the GWASs and the meta-GWAS datasets was conducted using R package SuperExactTest^28^, and the total number of genes was set to 30,000. The overlap network of five gene sets was plotted using Cytoscape^29^. Hypergeometric test was carried out to identify classes of genes that were overrepresented in an identified set of genes in the Molecular Signatures Database^30^.

### Estimation of heritability and genetic correlations

Partitioned heritability was carried out using stratified linkage disequilibrium score regression^31^. Stratified LD scores were calculated from the European-ancestry samples in the 1000 Genomes Project, and only the HapMap 3 SNPs were included with a MAF > 0.05.

The linkage disequilibrium score regression^32^ was used to indicate whether each dataset had sufficient evidence of a polygenic signal indicated by a *Z*-score > 4. Only SNPs that were in HapMap 3 with MAF > 0.05 in the 1000 Genomes European reference samples were included. Indels and structural variants were removed as strand-ambiguous variants. SNPs whose alleles did not match with those in the 1000 Genomes Project were excluded as well. LD scores and weights for use with European populations were downloaded from (http://www.broadinstitute.org/~bulik/eur_ldscores/).

We then used bivariate LD score regression to assess whether the results of meta-analysis have the same genetic architecture as the original GWAS, and calculated the genetic correlations amongst the five disorders.

To analyze the genetic structure of the five correlated psychiatric traits, GenomicSEM package^33^ was used to conduct genomic structural equation modeling (SEM) and exploratory factor analysis (EFA). Genetic covariance matrix (S) and sampling covariance matrix (V) for the five psychiatric disorders were estimated using multivariable LD score regression method. Genomic SEM can synthesize genetic correlations and SNP-heritabilities derived from GWAS summary statistics of individual traits with different and unknown degrees of overlap^33^. Indices of a fitting model include standardized root mean square residual (SRMR), Akaike information criterion (AIC), and comparative fit index (CFI).

### Protein–protein interactions (PPIs) and knowledge-based algorithms

PPIs mediate essentially biological processes, and PPI analyses have been previously performed in a number of complex diseases. After using GWAS to identify SNPs, PPI analyses have been undertaken to prioritize the genes most likely to be functionally significant to pathological mechanism^34,35^. It has been assumed that the identified genes will be involved in a common set of biological pathways or processes, which may be perturbed in the disease. In the current meta-study, PPI analysis was carried out using Disease Association Protein–Protein Link Evaluator (DAPPLE)^36^. The InWeb database compiles PPI data from a variety of sources, including molecular interaction, biomolecular interaction network database, and Kyoto encyclopedia of genes and genomes^36^.

Knowledge based algorithms were analyzed by the Pathway Studio database (www.pathwaystudio.com, accessed in March 2019)^37^. The Pathway Studio database contains over 11.8M unique associations supported by ≥40 million references.

### Polygenic risk score (PRS) for the summary results

PRS was calculated for the summary results of the ASD, ASD-MTAG, SCZ, and SCZ-MTAG using PRS-CS software^38^. GSE9222 dataset for ASD^39^ and GSE23201 dataset for SCZ^40^ were applied for validation of PRS.

## Results

### MTAG results

**Fig. 1:**
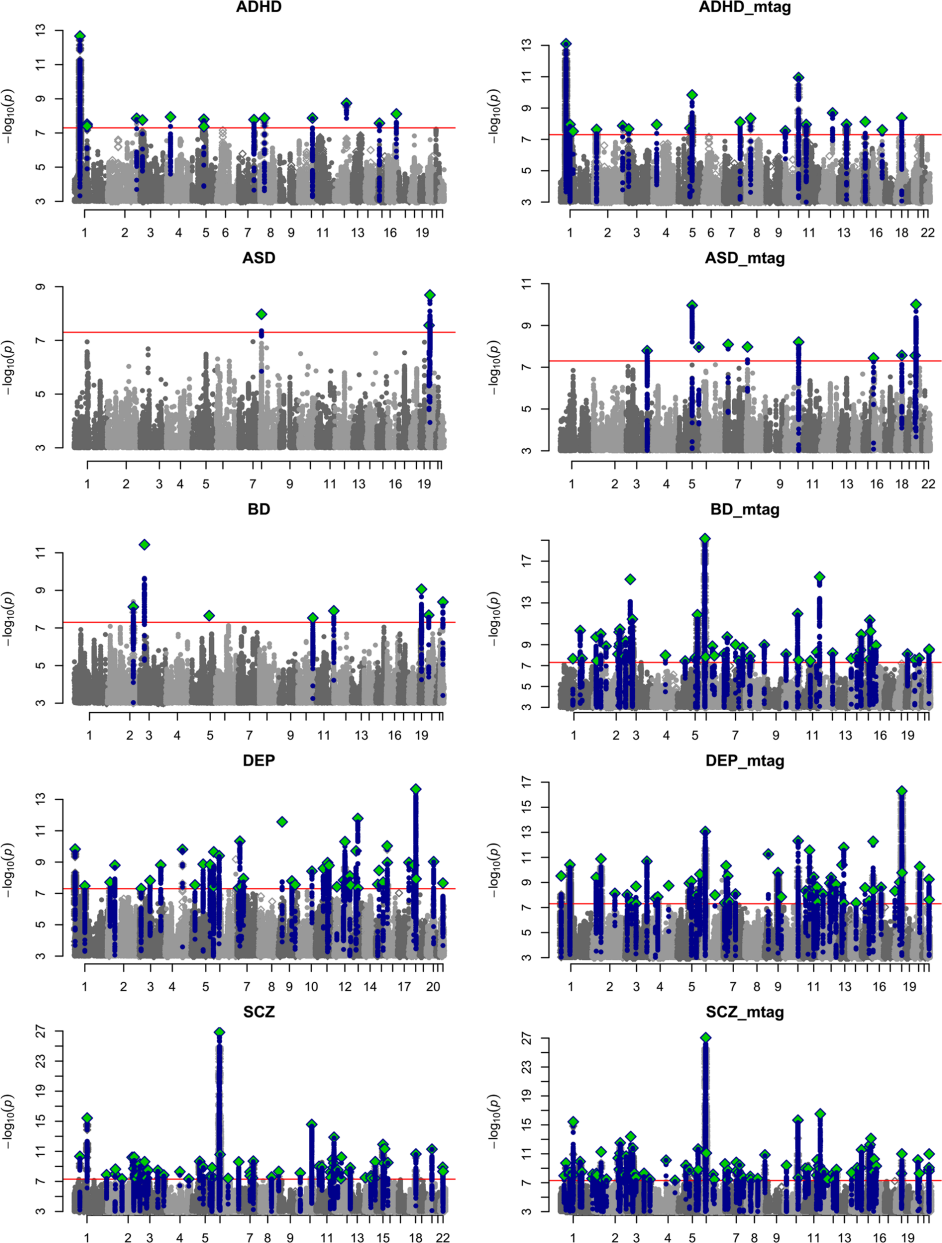
Manhattan plots of GWAS and MTAG results. The left and right panels display GWAS and MTAG results, respectively. The *X*-axis is the chromosomal position, and the *Y*-axis is negative log10 transformed *P*-values for each SNP. The red line indicates genome-wide significance (*P* = 5 × 10^–8^). Green diamonds indicate an independent genome-wide significant association (lead SNP). Blue points show SNPs in LD with lead SNPs.

Our single-trait input files are the results of previously published GWAS or GWAS meta-analyses. The schematic overview of the five cohorts is shown in the Supplementary figures and notes. Summary statistics of GWAS for ADHD, ASD, BD, DEP, and SCZ were analyzed (Fig. 1). The effective sample size in the MTAG results was estimated to be 61,421, 60,817, 65,682, 955,012, and 67,764 for ADHD, ASD, BD, DEP, and SCZ, respectively.

In addition, we compared the results of the original GWAS with those of MTAG. The summary of comparative analyzes of GWAS and MTAG results is shown in Table 1. The mean *χ*^2^ of the five disorders increased in MTAG results. The QQ plots and *λ*_GC_ of all GWAS and MTAG results are shown in Supplementary Fig 1. No evidence of inflation was found, as shown in Table 1. *λ*_GC_ values for ADHD, ASD, BD, DEP, and SCZ ranged from 0.91 to 1.05 in the GWAS results, and from 0.92 to 1.02 in the MTAG results.

**Table 1:** Statistical summary of GWAS and MTAG results.

	ADHD		ASD		DEP		BD		SCZ	
	GWAS	MTAG	GWAS	MTAG	GWAS	MTAG	GWAS	MTAG	GWAS	MTAG
SigSNPs	304	659	93	436	1890	3706	148	3343	8740	10609
Lead SNPs	15	21	3	10	49	63	8	63	87	110
Genomic locus	14	19	3	10	45	57	8	54	64	87
*χ*^2^	1.30	1.33	1.20	1.26	1.54	1.60	1.32	1.50	1.63	1.64
*h*^2^	0.23	0.33	0.19	0.35	0.04	0.05	0.35	0.70	0.42	0.51
Lambda GC	1.25	1.25	1.17	1.19	1.43	1.45	1.26	1.34	1.49	1.46
Intercept	1.03	0.95	1.01	0.92	1.01	0.98	1.02	0.89	1.05	0.95
Ratio	0.12	<0	0.04	<0	0.01	<0	0.05	<0	0.08	<0
Ngenes	20	13	43	121	254	24	17	160	136	269
Sample size	55,374	61,421	46,350	60,817	688,809	955,012	41,653	65,682	65,967	67,764
Overlapped SNPs	520,857		520,857		520,857		520,857		520,857	

*SigSNPs* number of significant SNPs; *Ngenes* number of genes.

After running the MTAG analysis, the number of lead SNPs increased from 15 to 21 in ADHD, from 3 to 10 in ASD, from 49 to 63 in DEP, from 8 to 63 in BD, and from 87 to 110 in SCZ. The number of genomic loci increased from 14 to 19 in ADHD, from 3 to 10 in ASD, from 45 to 57 in DEP, from 8 to 54 in BD and from 64 to 87 in SCZ (Table 1). The detailed information is summarized in Supplementary Tables S1, S2.

### Biological annotation

**Fig. 2:**
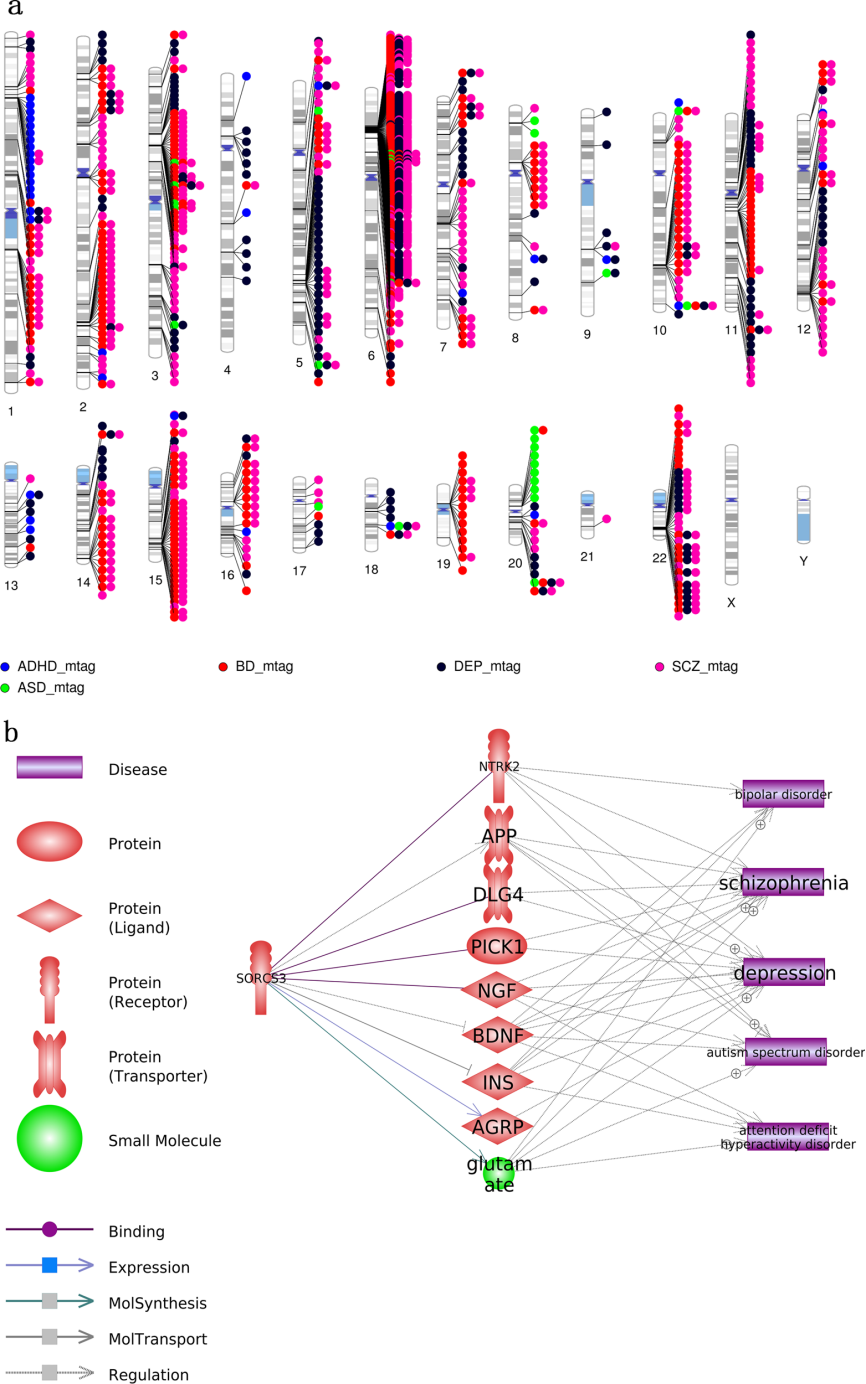
Significant genes and SORCS3’s correlation with the five disorders. **a** Chromosomal ideograms for significant genes of MTAG results. Chromosomal locations containing significant genes of different phenotypes are annotated in different colors. The phenotype circles were arranged in a proximity algorithm. **b** Biological pathways between the *SORCS3* gene and the five disorders.

We analyzed both GWAS and MTAG results using FUMA. Summary of input and background genes is presented in Supplementary Tables S3, S4. Manhattan plots of the gene-based test as computed by MAGMA based on GWAS and MTAG summary statistics are depicted in Supplementary Fig. 2. The results of significant genes and enriched gene sets in MAGMA are presented in Supplementary Tables S5, S6). Chromosomal ideograms for significant genes in MAGMA of GWAS results and MTAG are illustrated in Fig. 2. We also summarized the unique genes and overlapped genes in MAGMA of each panel (Supplementary Table S7). There was an overrepresentation of SNPs annotated in intergenic and intronic non-coding RNAs (Supplementary Fig. 3).

To evaluate the significance of tissue types of specific expression, tissue specificity analysis was conducted by MAGMA. MAGMA was performed using the result of gene analysis (gene-based *P* value) and tested for one side (greater) with conditioning on average expression across general tissues in total taken from the GTEx v6 RNA-seq database. Genes with *P* value ≤ 0.05 after Bonferroni correction and absolute log fold change ≥0.58 were defined as differentially expressed genes compared with the given label. Results of the tissue enrichment analysis based on the GWAS and MTAG results are displayed in Supplementary Fig. 4 and Table S8. Almost all the enrichment of the signals was found in the brain and pituitary.

### Genetic correlation

**Fig. 3:**
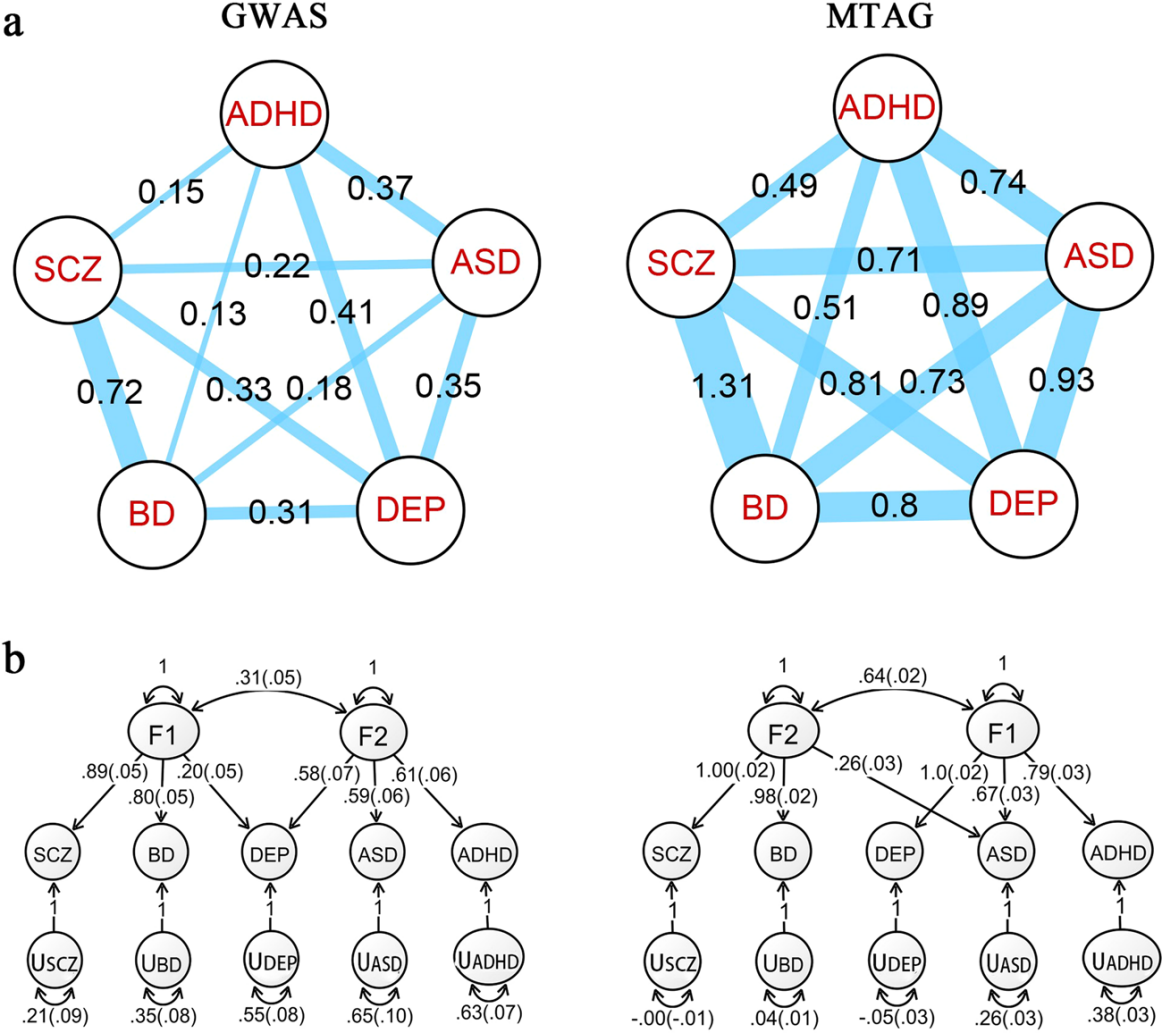
Genetic correlations and path diagram of genomic structural equation modeling (SEM) of the five disorders. The left and right panels display GWAS and MTAG results, respectively. **a** Genetic correlations between five psychiatric disorders. Line width is proportional to genetic correlations between five psychiatric disorders. The numbers show *r*_g_s amongst the five disorders. **b** Path diagram of genomic SEM and exploratory factor analysis (EFA) analyses of the five disorders in the GWAS and MTAG results. The values were standardized results of confirmatory factor analysis.

Genetic correlation is the proportion of variance at genetic level, indicating the degree of pleiotropy and genetic overlap. We analyzed the genetic correlation between each pair of disorders. The correlation between the GWAS datasets, and the correlation between the MTAG datasets are shown in Fig. 3a, Supplementary Fig. 5, and Supplementary Table S9. The results showed that each of the GWAS datasets had high *r*_g_ (≥0.77) with corresponding MTAG datasets, indicating that there was no a significant difference between them. In the GWAS results, the genetic correlation was high between SCZ and BD (0.72), moderate between ADHD and DEP (0.41), ADHD and ASD (0.37), ASD and DEP (0.35), SCZ and DEP (0.33), BD and DEP (0.31), and low between other pairs of disorders. However, in MTAG results, *r*_g_ of all pairs of disorders noticeably increased^19^.

Genomic SEM and EFA were applied to analyze the five disorders in the GWAS and MTAG results. Genomic SEM and EFA analyses indicated a two-factor model, therefore, we fitted the two-factor model in Genomic SEM using a form of confirmatory factor analysis. According to our results, the five disorders were clustered into two groups of inter-related disorders for both the GWAS results and the MTAG results, with SCZ and BD were involved in a group and the other three disorders were in another group. The model fit was adequate for both the GWAS results (*χ*^2^(3) = 7.24, AIC = 31.2, CFI = 1.00, SRMR = 0.022) and the MTAG results (*χ*^2^(3) = 5,296,998, AIC = 5,297,022, CFI = −16.0, SRMR = 0.017). Standardized results of confirmatory factor analysis were presented using path diagram (Fig. 3b).

### Gene identification and gene-overlap analysis

We compared the overlaps of genes among the five gene sets of the GWAS with those of the meta-GWAS datasets. The significant genes in GWAS and MTAG are listed in Supplementary Table S4. In the GWAS, remarkable overlaps were observed in most pairs of disorders. There were 123 cross-trait genes, including 120 genes common in two disorders and three genes common in three disorders. *GABBR1* was implicated in SCZ, DEP, and ASD (odds ratio (OR) = 464.8, Fisher’s *P* = 0.002). SORCS3 and LINC00461 were shared by ADHD, DEP, and SCZ, which did not likely occur by chance (OR = 641.1, Fisher’s *P* = 4.66 × 10^−6^).

In the MTAG results, there were notable overlaps between each pair of disorders, except for the pair between ADHD and BD. There were a total of 366 cross-trait genes, including 220, 138, 7, and 1 genes common in 2, 3, 4, and 5 disorders. Most strikingly, SORCS3 was the 5-trait gene (OR = 14292790, Fisher’s *P* = 7.00 × 10^−8^). The detailed data can be found in Supplementary Table S10.

In addition, PPI analysis was conducted using DAPPLE for prioritized genes in each disorder. DAPPLE looks for significant physical connections between proteins encoded by disease-related genes in loci, based on PPIs reported in the literature^36^. The PPI networks show significant direct and indirect connectivity. Detailed information including each *P* value is shown in Supplementary Table S11. These results indicated that the networks are more densely connected. The number of direct and indirect connectivity of all diseases (except for ADHD) increased in MTAG, as well as in PPI networks.

### Knowledge-based algorithms

**Fig. 4:**
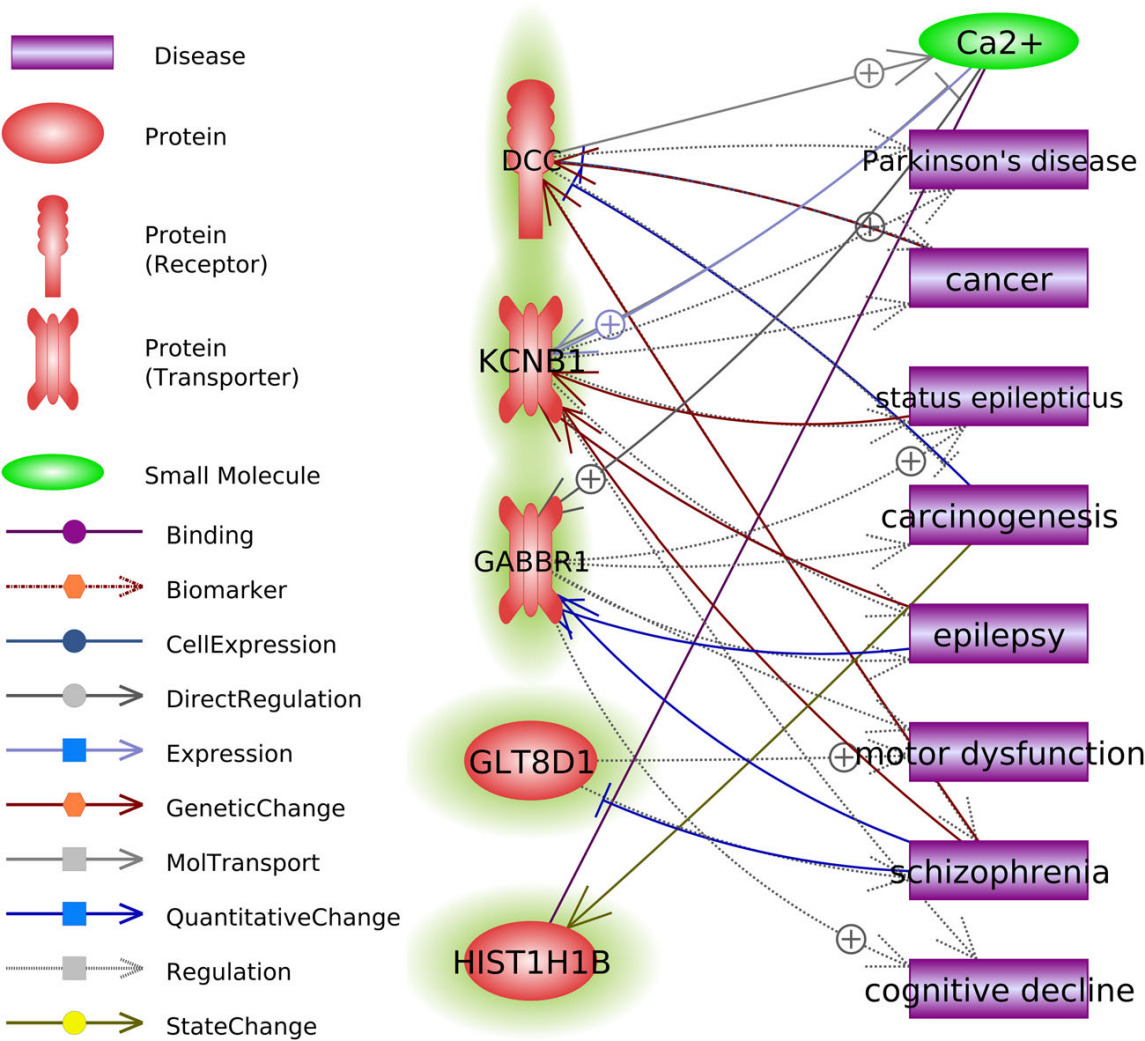
Sub-network enrichment analysis (SNEA) results of the eight genes. Each of the item identified from SNEA associated with at least two common genes.

Literature evidence indicated that *SORCS3* is correlated with the five disorders through eight proteins (NGF, APP, DLG4, PICK1, INS, BDNF, AGRP, and NTRK2) and a small molecule glutamate (Fig. 2b and Supplementary Table S12). Knowledge-based gene-trait correlations between the other seven highly pleotropic genes and the five disorders are displayed in Supplementary Figs. 6–10 and Supplementary Tables S12, S13. We also conducted a sub-network enrichment analysis to explore diseases or coupons as common targets connecting the eight genes common in four or five disorders. Results showed that five out of the eight genes were linked to common diseases/coupons (Fig. 4), which supported the functional association among these genes.

### PRSs of the summary results

The valid samples for ASD included 426 unrelated probands and 232 parents (116 trios). A total of 258,000 valid markers were remained in the GSE9222 dataset after quality control. Nearly 142,000 markers were overlapped between the GSE9222 dataset and the PRS. For ASD, the mean value of total PRS was −0.0026 and 0.0014 for controls and cases, respectively. For ASD_MTAG, the mean value of total PRS was −0.0040 and 0.0022 for controls and cases, respectively. The area under the receiver operating curve (AUC) was 0.525 and 0.543 for ASD (sensitivity, 0.510; specificity, 0.515) and ASD_MTAG (sensitivity, 0.517; specificity, 0.526), respectively. Although the performance of the predictions was not optimal, it was unveiled that the MTAG results outperformed compared with original ASD results.

The valid samples for SCZ included 1044 patients and 2052 controls. A total of 1,016,000 valid markers were remained in this dataset after quality control. Nearly 123,000 valid markers were overlapped between the GSE9222 dataset and the PRS. For SCZ, the mean value of total PRS was −0.027 and 0.053 for controls and cases, respectively. For SCZ_MTAG, the mean value of total PRS was −0.034 and 0.067 for controls and cases, respectively. The AUC was 0.832 and 0.811 for SCZ (sensitivity, 0.721; specificity, 0.612) and SCZ_MTAG (sensitivity,0.706; specificity, 0.604), respectively (Supplementary Table S14). The performance of the original GWAS results and that of the MTAG results was found comparable.

## Discussion

The present meta-analysis confirmd that there were varied degrees of genetic associations among the five major psychiatric disorders, particularly between SCZ and BD. A high genetic correlation between SCZ and BD and a moderate genetic correlation between ADHD and DEP observed in our study were consistent with previous reports. However, our results indicated a moderate genetic correlation between ADHD and ASD, ASD and DEP, which was inconsistent with the literatures^19,20^, possibly because a great number of samples for ASD were employed in our study (*n* = 46350) compared with Lee et al.^19^ (*n* = 6731) and Brainstorm et al.^20^ (*n* = 7377). Our results uncovered a moderate genetic correlation among ADHD, ASD, and DEP. The Genomic SEM also suggested that these three disorders belonged to the same group.

The substantial genetic correlations among psychiatric disorders indicated that they could not be regarded as completely independent disease entities, and cross-disorder analysis will be the direction of future studies. Meanwhile, as evidenced by the results of our study, the five mental disorders have their own unique pathogenic genes besides the shared genes, which provide further support for the change of DSM-5 diagnostic criteria. For example, bipolar and related disorders have been separated from the depressive disorder and placed between the chapters on SCZ spectrum and other psychotic disorders and depressive disorders, as a bridge between the two diagnostic classes in terms of clinical manifestations (see DSM-5, American Psychiatric Association), family history^15^ and genetics. Our study provides novel evidence supporting the relatedness of ASD, ADHD, and DEP. Taken together with several phenotypic studies (neuroimaging^41^, cognitive function^15^, and clinical phenotypic studies^16^), our study provides new evidence for the RDoC for mental disorders.

In both the GWAS and the MTAG datasets, we detected a constellation of cross-trait genes and the enrichment of common genes between most (for the GWAS datasets) or all (for the MTAG datasets) pairs of disorders, providing a further support for the genetic correlation among these disorders. We also observed significant enrichment of common genes among three or more disorders, and these genes may be taken as credible common genes into consideration for the five disorders. In the MTAG results, a total of 146 genes were observed for three or more disorders, including some genes that were previously known to be involved in psychosis, such as *DRD2* and *TCF4*, which were originally reported in SCZ and DEP, while our data indicated their association with BD.

Among 146 genes, there were seven genes common to four disorders, including *GLT8D1*, *KCNB1*, *GABBR1*, *HIST1H1B*, *HIST1H2BN*, and *HIST1H4L* in SCZ, DEP, BD, and ASD, and *DCC* in ADHD, ASD, DEP, and SCZ. Previous studies have documented the direct or indirect biological relationship between these genes and the five disorders (Supplementary Figs. 6–10 and Supplementary Table S12). The *GABBR1* gene encodes a neurotransmitter receptor for gamma-aminobutyric acid (GABA), the main inhibitory neurotransmitter in the central nervous system. *GABBR1* has been reported to be involved in SCZ^42^ and DEP^21^. The *KCNB1* gene encodes a voltage-sensing α-subunit of a delayed rectifier potassium channel. *KCNB1* is expressed in various nerve cells in the brain and can regulate neuronal excitability^43,44^. *DCC* plays a pivotal role in axon guidance and nerve regeneration^45^. In addition to its association with the four disorders, *DCC* is also a genome-wide candidate gene for intelligence^46^, cognitive ability^47^, and educational attainment^47^.

*HIST1H1B*, *HIST1H2BN*, and *HIST1H4L* are three genes of the seven genes associated with the four disorders. *HIST1H1B*, also known as linker histone H1.5, influences mRNA splicing and DNA binding and is involved in regulation of splice site selection and alternative splicing. *HIST1H2BN* and *HIST1H4L* belong to cluster histone involved in DNA binding. Histone is the main protein component of chromatin and plays a role in gene regulation. In the GO, these three genes were found to be enriched in 63 pathways, including nucleosome. Histone modifications play fundamental roles in several biological processes that are involved in the manipulation and expression of DNA^48^. Histone modifications act in gene regulation, including lysine acetylation. It may indicate the epigenetic variation in psychiatric disorders.

The most striking result obtained from the *SORCS3* (Sortilin Related VPS10 Domain Containing Receptor 3) gene. In the original GWAS results, *SORCS3* was shown to be associated with ADHD, DEP, and SCZ; however, this meta-analysis provided additional support for involvement of BD and ASD as well. *SORCS3* showed to have a myriad of functional partners, including NGF, APP, DLG4, PICK1, INS, BDNF, AGRP, and NTRK2, which may mediate the association of *SORCS3* with the five disorders (Fig. 2b and Supplementary Table S12).

The *SORCS3* gene encodes a type-1 receptor transmembrane protein that is a member of the vacuolar protein sorting 10 receptor family. VPS10P (vacuolar protein sorting 10 protein)-domain receptors are sorting receptors that control the intracellular trafficking of target proteins in neurons^49,50^. *SORCS3* binds nerve growth factor (NGF) and platelet-derived growth factor (PDGF-BB)^51,52^. The expression of *SORCS3* is almost restricted to the brain and spinal cord^53^, and its expression in hippocampus is induced by neuronal activity^54,55^. *SORCS3*, together with another VPS10p-domain receptor-SORCS1, can control energy balance and orexigenic peptide production by attenuating *BDNF* signaling^56^. *SORCS1* is a stronger regulator of glutamate receptor functions compared with GABAergic signaling, thereby acting as a key regulator of synaptic transmission and plasticity^57^. *SORCS3* is also a postsynaptic modulator of synaptic depression and fear extinction, affecting *NMDA* receptor-dependent and -independent long-term depression^58^.

The role of *SORCS3* has extended beyond the five disorders. *SORCS3* has been reported to be genome-widely associated with several other brain relevant phenotypes, including intelligence^46^, cognitive ability^47^, educational attainment^47^, subjective well-being^9^, and neuroticism^9^. *SORCS3* has also been reported to be associated with Alzheimer’s disease^59,60^. Although S*ORCS3* is genome-widely implicated in multiple neurodevelopment-related traits, its role has not received enough attention in each trait.

We assessed a hypothesis that the more times a gene is observed across different traits, the more likely the gene is a solid risk gene for each trait involved rather than a stochastic noise, and the more likely the gene can represent shared genetic etiology of different traits. In general, all theabove-mentioned eight genes play a role in neurodevelopment in human, which is consistent with pathogenic mechanism of the mental disorders. In addition, an evidence indicates that these gene may be functionally inter-correlated, since five out of these eight genes are linked to common diseases, including cancer, carcinogenesis, and several brain-related conditions (Parkinson’ disease, epilepsy, motor dysfunction, cognitive impairment and SCZ). In addition to confirming several previous cross-traits genes (e.g., *DRD2* and *TCF4*), the present study highlighted several new ones, such as *SORCS3*, *DCC*, *GLT8D1*, *GABBR1*, and *KCNB1*. These genes may be risk genes with pleiotropic roles that partially underlie the common genetic architecture of multiple psychiatric disorders. Together, these findings may shed light on the molecular mechanism of the major psychoses.

In PPI analyses, we identified significant direct and indirect connectivity and interrelated genes/proteins for the five mental disorders. Our study achieved more significant results and larger networks with more nodes and edges by using the genes from MTAG compared with the genes from GWAS only, indicating a greater statistical power. Thus, it can be concluded that the PPI network generated by MTAG results can better reflect the underlying pathological mechanism of mental disorders and expand our understanding about overlapping mechanisms among different mental disorders.

One of the potential limitations of our study is that the MTAG results are susceptible to bias and a high FDR during analysis of sets of GWAS summary statistics where some are remarkably high powered than others^9^. Another limitation is that we failed to achieve individual genotypes, which resulted in the incompleteness of some important analyses, such as PRS, and so on.

In conclusion, our study confirmed the substantial genetic correlations among the five mental disorders and revealed common genes among these disorders, aiding in understanding their genetic structure and pathophysiological mechanisms.

## Supplementary information

Supplementary Material

Supplementary Table S1

Supplementary Table S2

Supplementary Table S3

Supplementary Table S4

Supplementary Table S5

Supplementary Table S6

Supplementary Table S7

Supplementary Table S8

Supplementary Table S9

Supplementary Table S10

Supplementary Table S11

Supplementary Table S12

Supplementary Table S13

Supplementary Table S14

## Data Availability

All data generated or analyzed in this study are included in the published articles (see References).
