# Correction: Multi-trait analysis for genome-wide association study of five psychiatric disorders

**DOI:** 10.1038/s41398-020-00924-0

**Published:** 2020-07-14

**Authors:** Yulu Wu, Hongbao Cao, Ancha Baranova, Hailiang Huang, Sheng Li, Lei Cai, Shuquan Rao, Minhan Dai, Min Xie, Yikai Dou, Qinjian Hao, Ling Zhu, Xiangrong Zhang, Yin Yao, Fuquan Zhang, Mingqing Xu, Qiang Wang

**Affiliations:** 1 Mental Health Center and Psychiatric Laboratory, State Key Laboratory of Biotherapy, West China Hospital of Sichuan University, Chengdu, Sichuan China; 2 West China Brain Research Center, West China Hospital of Sichuan University, Chengdu, Sichuan China; 3 Department of Psychiatry, First Clinical Medical College/First Hospital of Shanxi Medical University, Taiyuan, China; 4 School of Systems Biology, George Mason University (GMU), Fairfax, VA USA; 5 Research Centre for Medical Genetics, Moscow, Russia; 6 Analytic and Translational Genetics Unit, Massachusetts General Hospital, Boston, MA USA; 7 Stanley Center for Psychiatric Research, Broad Institute of Harvard and MIT, Cambridge, MA USA; 8 Bio-X Institutes, Key Laboratory for the Genetics of Developmental and Neuropsychiatric Disorders (Ministry of Education), Shanghai Jiaotong University, 1954 Huashan Road, Xuhui, 200030 Shanghai, China; 9 School of Life Science and Engineering, Southwest Jiaotong University, Chengdu, China; 10 The Center of Gerontology and Geriatrics, West China Hospital of Sichuan University, Chengdu, Sichuan China; 11 Department of Geriatric Psychiatry, Nanjing Brain Hospital, Affiliated to Nanjing Medical University, Nanjing, China; 12 Collaborative Innovation Center of Genetics and Development, School of Life Sciences, Fudan University, Shanghai, China; 13 Department of Psychiatry, The Affiliated Brain Hospital of Nanjing Medical University, 264 Guangzhou Road, Nanjing, China; 14 Shanghai Key Laboratory of Psychotic Disorders, Shanghai Mental Health Center, Shanghai Jiao Tong University School of Medicine, 600 South Wanping Road, Xuhui, 200030 Shanghai, China

Correction to: *Translational Psychiatry*

10.1038/s41398-020-00902-6 published online 11 July 2020

The original version of this Article was updated shortly after publication to correct the order of the corresponding authors. The correct order should be: Fuquan Zhang, Mingqing Xu and Qiang Wang.

