# Supplementary material for: Multi-trait analysis for genome-wide association study of five psychiatric disorders
Source: Transl Psychiatry. 2020 Jun 30;10:209. doi: 10.1038/s41398-020-00902-6 (PMC7326916; doi:10.1038/s41398-020-00902-6)
Supplement: Supplementary file 1 — Supplementary Material [file 41398_2020_902_MOESM1_ESM.docx]

SUPPLEMENTARY MATERIAL

**Table of contents**

Introduction

Supplementary figures

Figure 1 Quantile-quantile (QQ) Plots

Figure 2 Mahattan Plot (gene-based test)

Figure 3 Functional consequences of SNPs on genes

Figure 4 MAGMA Tissue Expression Analysis

Figure 5 Heatmap for gene correlation

Figure 6 Biological pathways between DCC and 4 traits

Figure 7 Biological pathways between GABBR1 and 4 traits

Figure 8 Biological pathways between GLT8D1 and 4 traits

Figure 9 Biological pathways between HIST1H1B, HIST1H2BN

and 4 traits

Figure 10 Biological pathways analysis between KCNB1 and 4 traits

Supplementary note 1 Information for the data

Supplementary note 2 Information for the DEP sample

Supplementary note 3 Introduction for FUMA

**Introduction**

This file contains detailed description about additional information for the DEP sample and introduction for FUMA. Supplementary figures and some of tables are also included in this file.


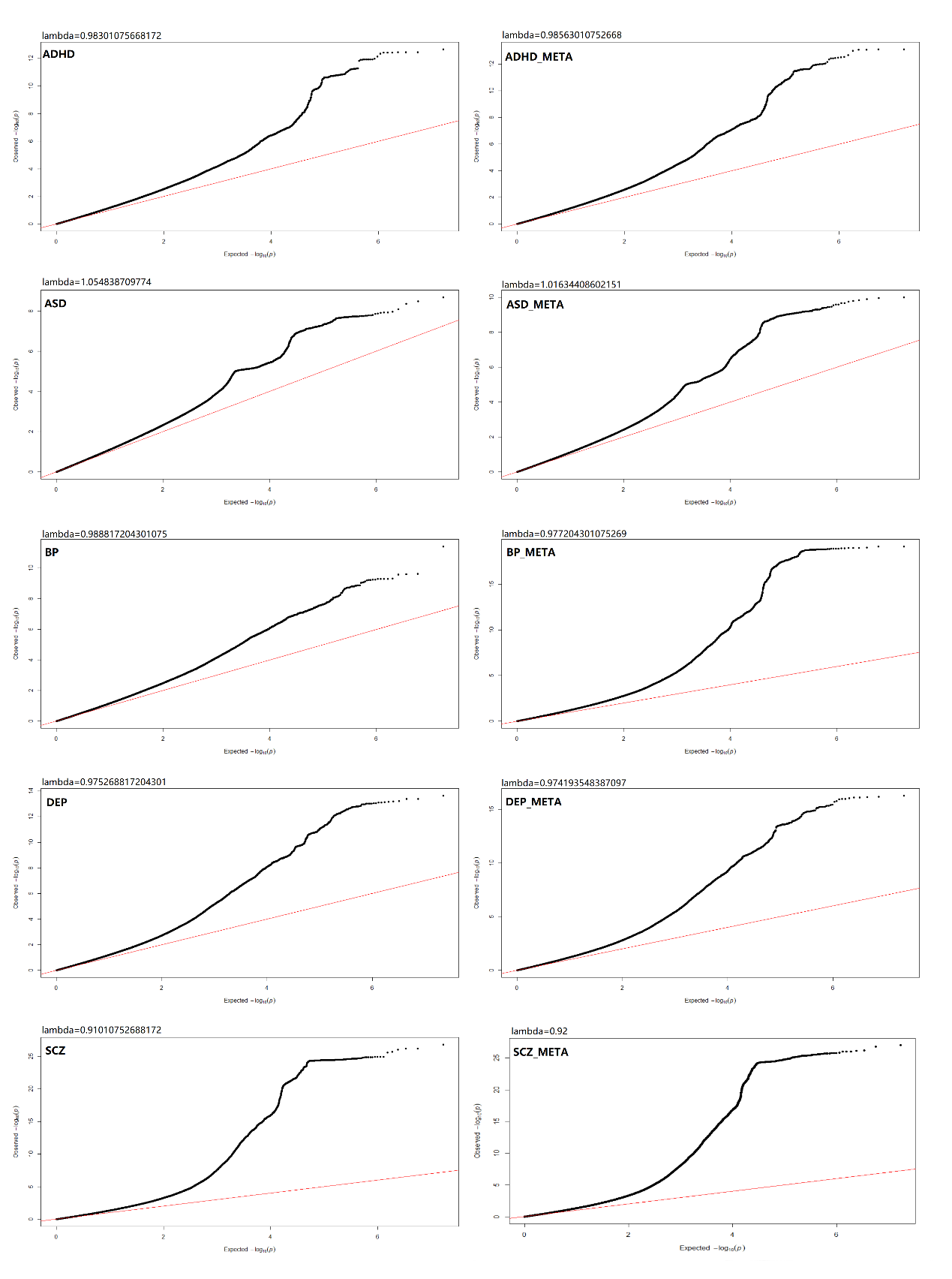


**Supplementary Figure 1. Quantile-quantile (QQ) Plots for GWAS and MTAG results.** λGC of each for the five analyses and MTAG results are shown in the pictures. All show strong evidence of no inflation. λGC for ADHD and MTAG result of ADHD are 0.98 and 0.99 respectively. λGC for ASD and MTAG result of ASD are 1.05 and 1.02 respectively. λGC for BD and MTAG result of BD are 0.99 and 0.98 respectively. λGC for DEP and MTAG result of DEP are 0.98 and 0.97 respectively. λGC for SCZ and MTAG result of SCZ are 0.91 and 0.92 respectively.


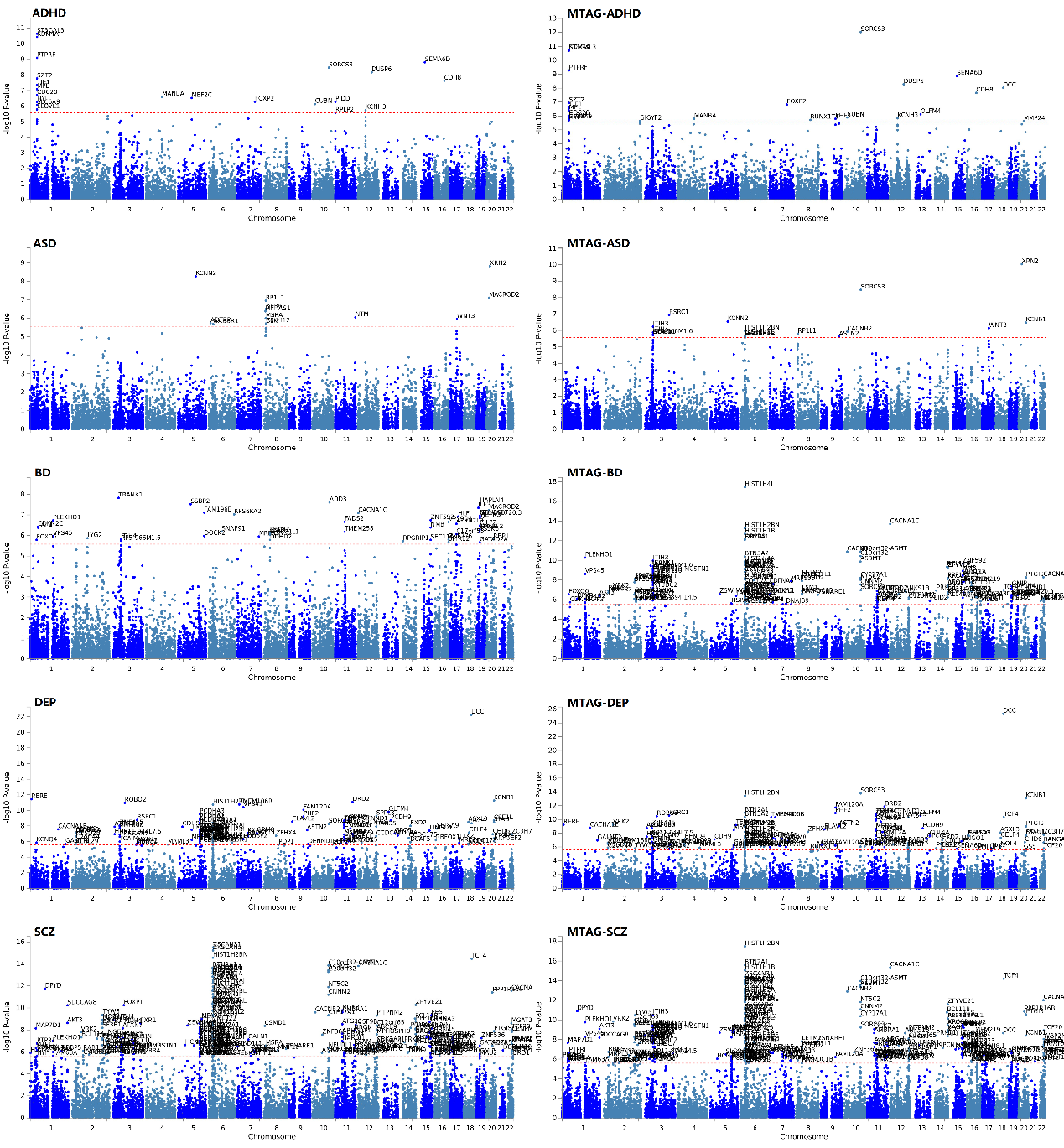


**Supplementary Figure 2. Mahattan Plot (gene-based test)** Manhattan plots of the gene-based test as computed by MAGMA based on GWAS and MTAG summary statistics. SNPs were mapped to 18770 protein coding genes. The dotted red line indicates the threshold for genome-wide significance (p<2.66×10-6). Genes reach genome-wide significance were labelled with their names.


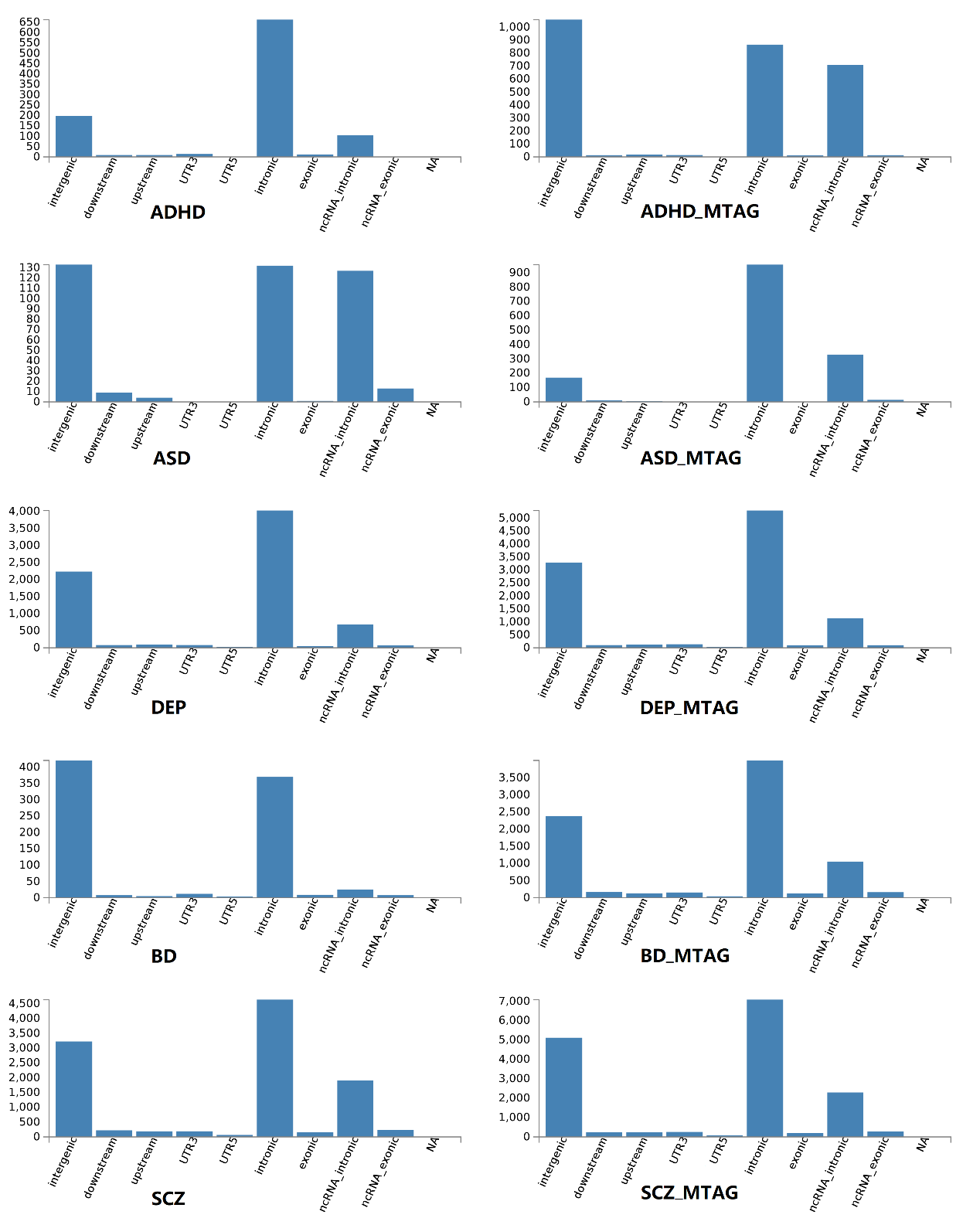


**Supplementary Figure 3. Functional consequences of SNPs on genes** This analysis was implemented in FUMA. In the histogram, the numbers of all SNPs in LD of lead SNPs which have corresponding functional annotations assigned by ANNOVAR. When SNP has one more annotation, each annotation is counted.


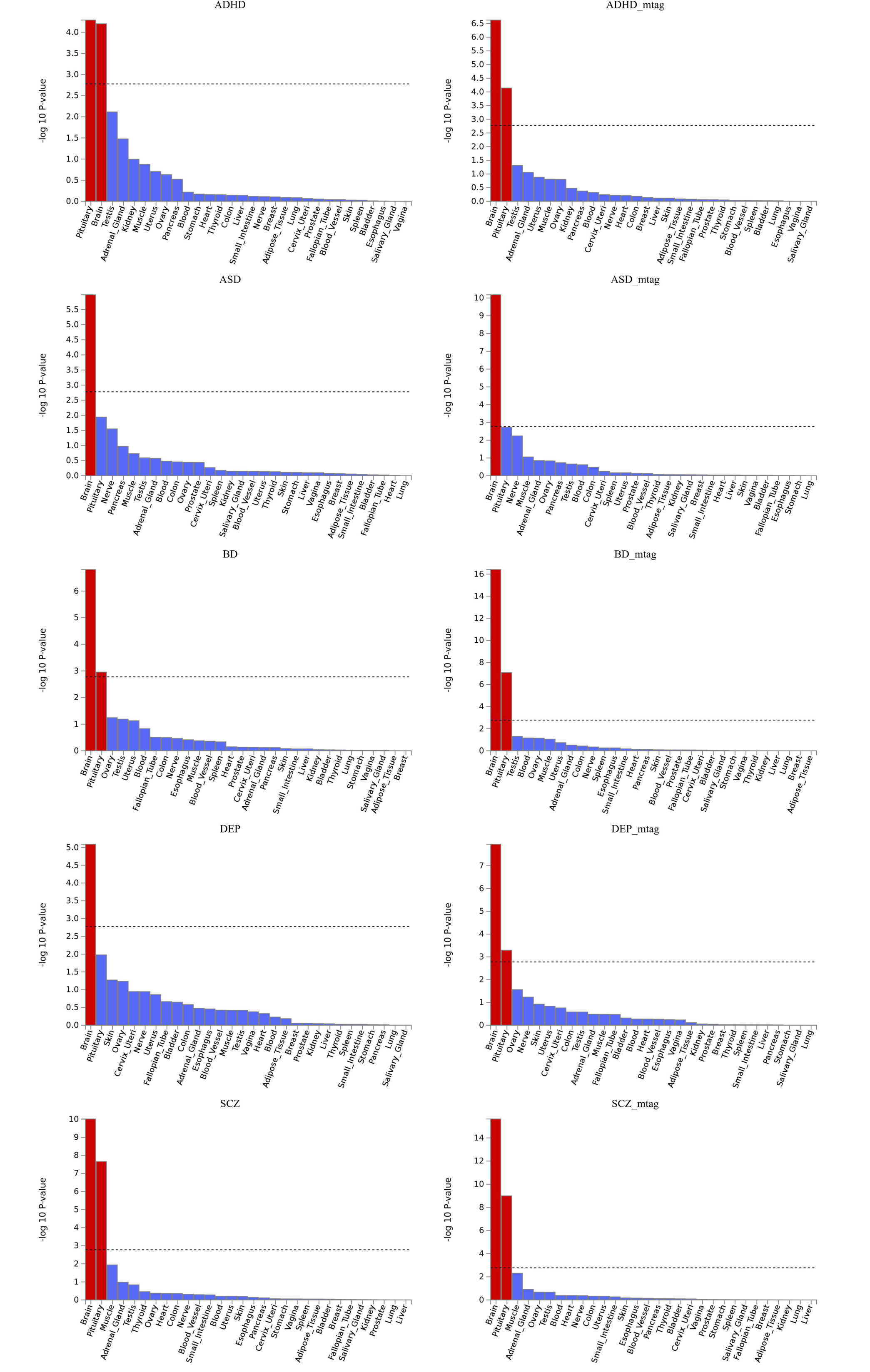


**Supplementary Figure 4. MAGMA Tissue Expression Analysis** MAGMA Tissue Expression Analysis implemented in FUMA. To test the (positive) relationship between highly expressed genes in a specific tissue and genetic associations, gene-property analysis is performed using average expression of genes per tissue type as a gene covariate. Gene expression values are log2 transformed average RPKM per tissue type after winsorized at 50 based on GTEx RNA-seq data. Tissue expression analysis is performed for 30 general tissue types and 53 specific tissue types separately. MAGMA was performed using the result of gene analysis (gene-based P-value) and tested for one side (greater) with conditioning on average expression across all tissue types. The dotted line indicates the Bonferroni-corrected α level, and the tissues that meet this significance threshold are highlighted in red.


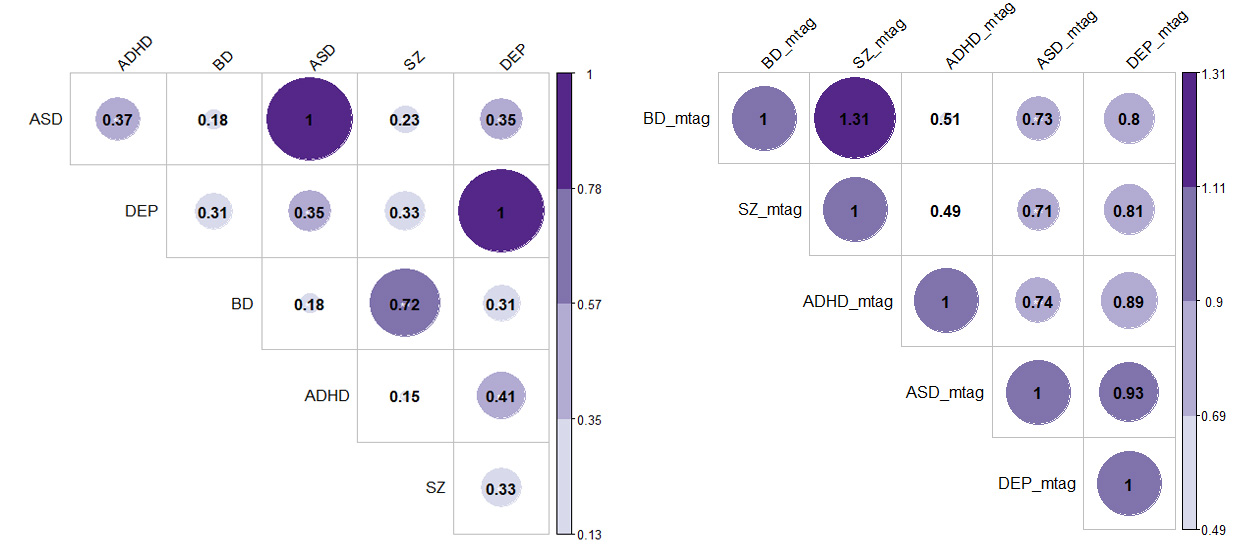


**Supplementary Figure 5.** **Heatmap for gene correlation of GWAS result and that after MTAG.** The figures were plotted by R package corrplot. These figures visualize the correlation matrix of GWAS result and MTAG result respectively. The colors of circles and the numbers show rg between each phenotype. The size of circles shows the p values of the corresponding correlation.

**Supplementary note for figure 6-10**

Supplementary figure 5-10 show biological pathways between the seven genes and the four traits. We compared the overlaps of genes among the 5 gene sets of the GWAS datasets with those of the 5 gene sets of the meta-GWAS datasets. The significant genes were converted into official gene symbols using R package limma. Genes that could not be mapped to any official gene symbols were removed. R package SuperExactTest was used for analysis of overlaps gene sets. The correlation network of 5 gene sets was plotted using Cytoscape. Hypergeometric tests were performed to identify classes of genes that were overrepresented in an identified set of genes in the Molecular Signatures Database. GABBR1, GLT8D1, HIST1H1B, HIST1H2BN, KCNB1, and DCC were common to 4 traits, including DEP, ASD, SCZ and BD. In each figure, red and green icons represent different kind of proteins and purple labels represent the diseases. Lines represent correlations between protein and protein or protein and disease.


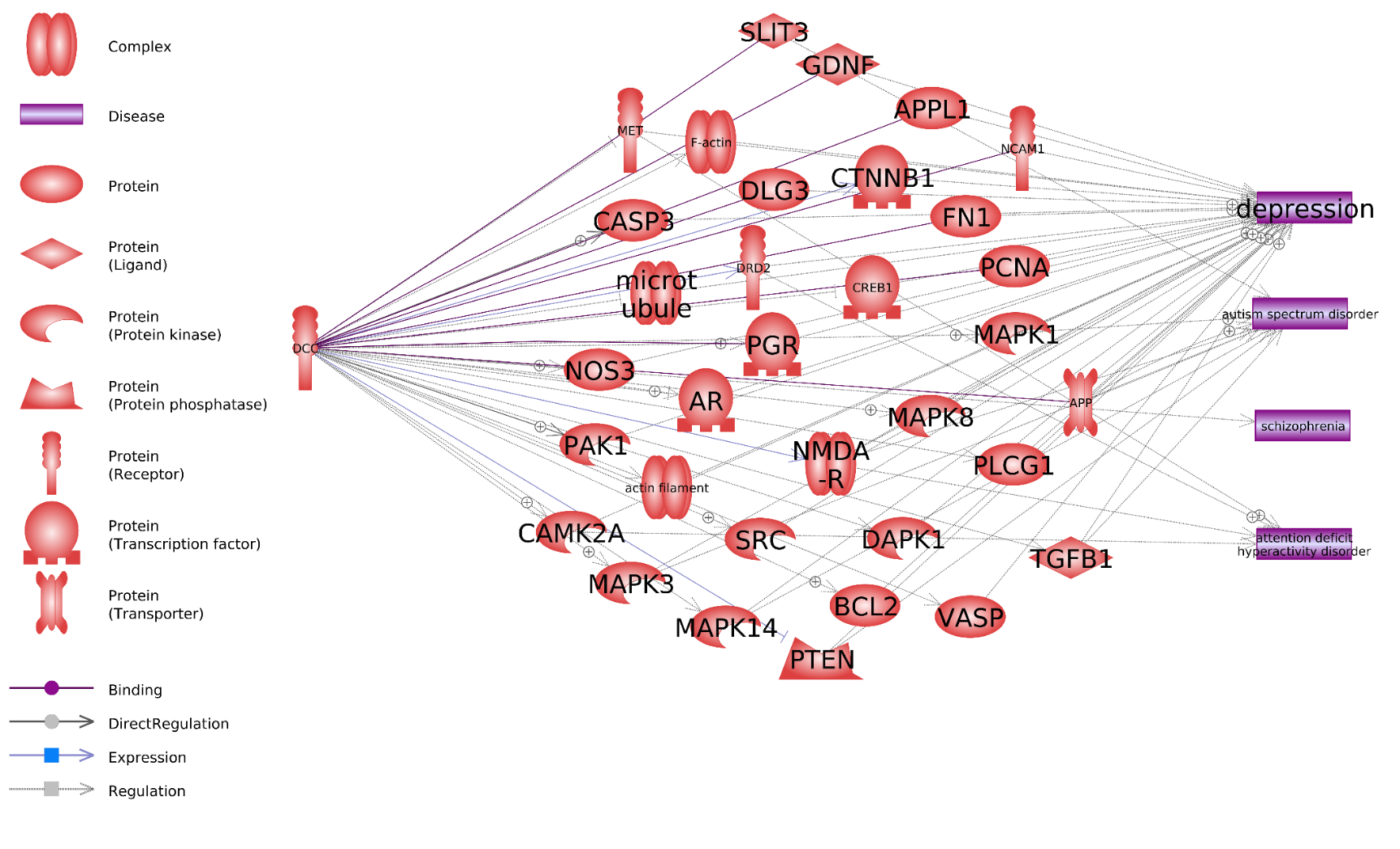


**Figure 6 Biological pathways between DCC and 4 traits.**


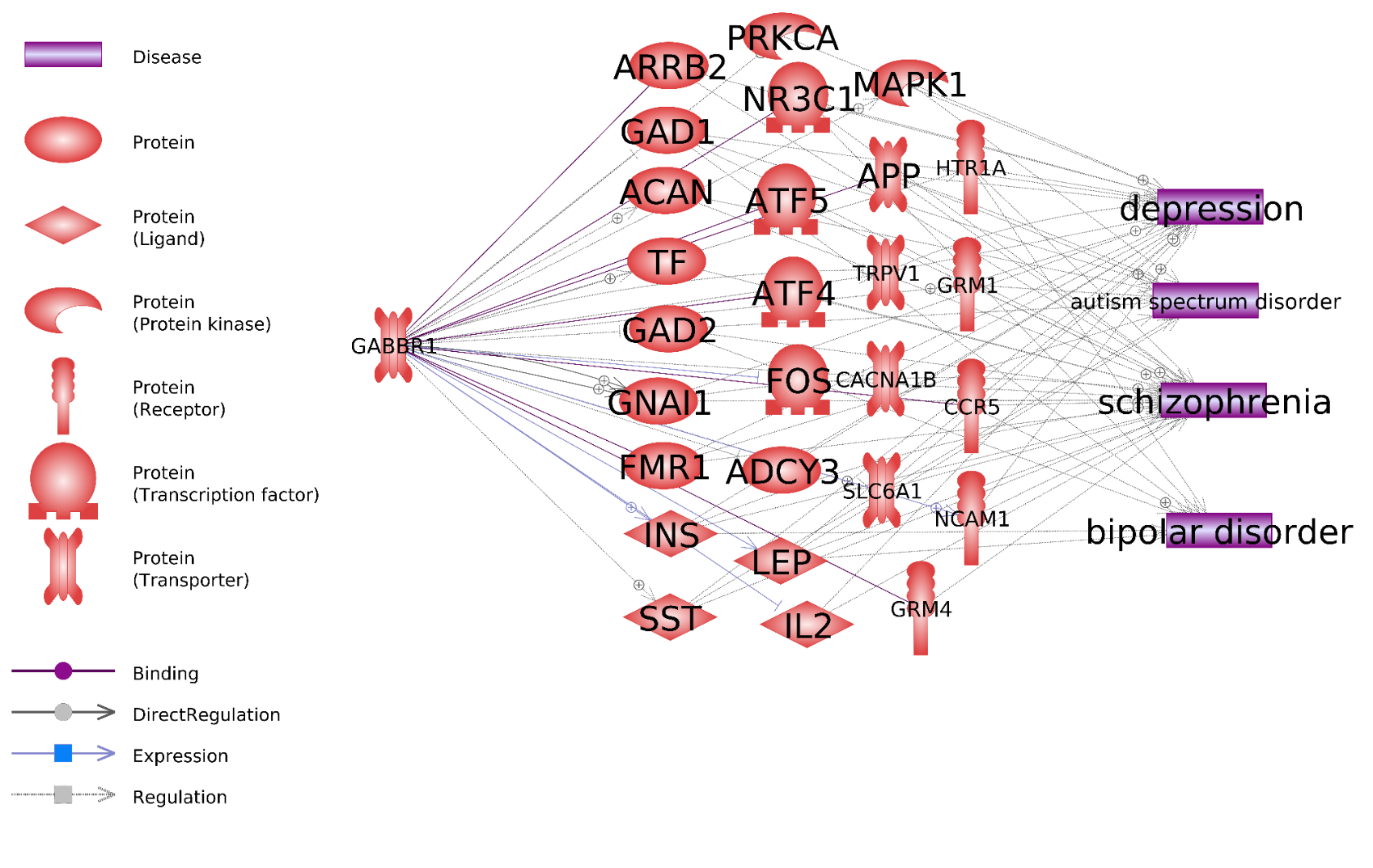


**Figure 7 Biological pathways between GABBR1 and 4 traits**


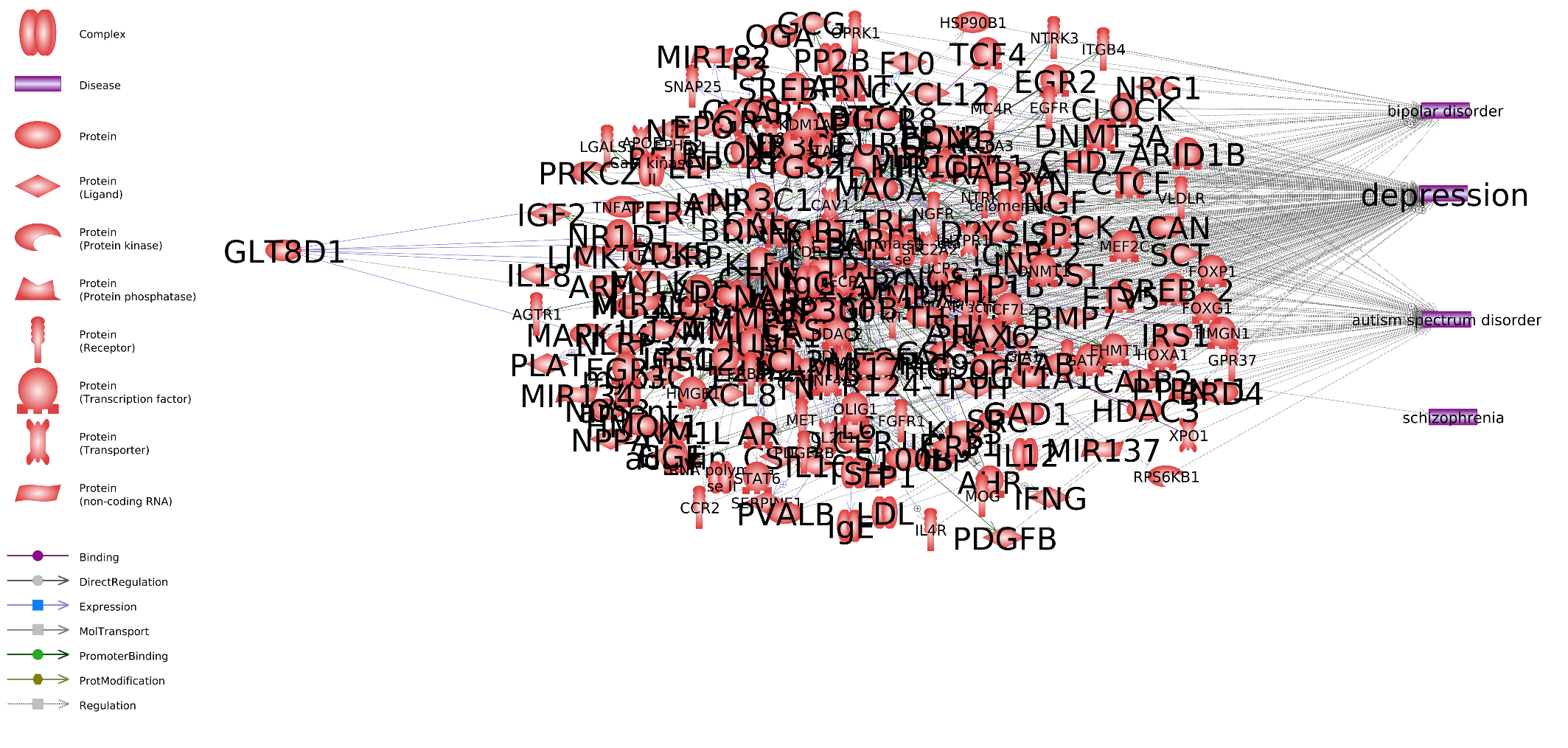


**Figure 8 Biological pathways between GLT8D1 and 4 traits**


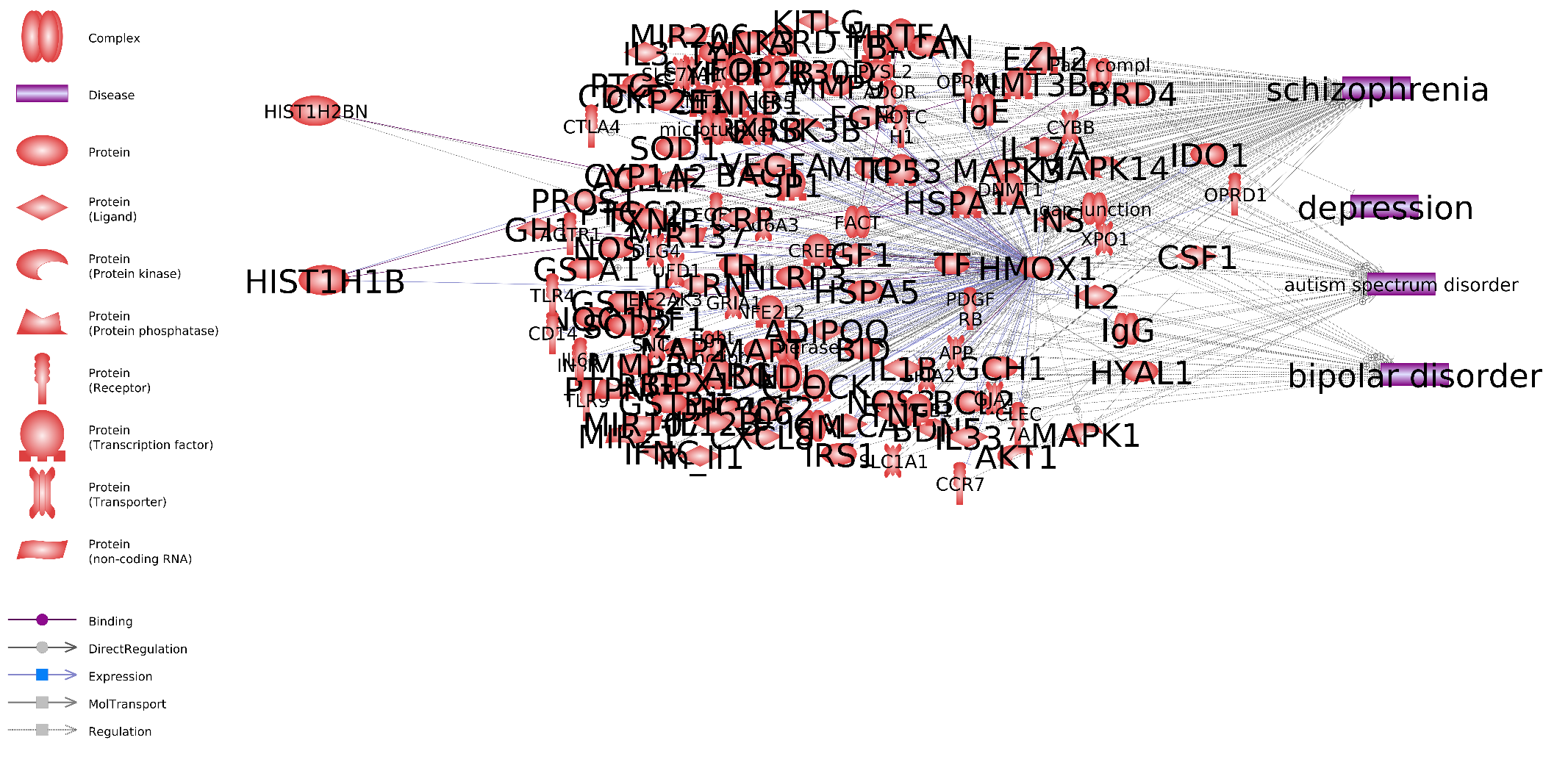


**Figure 9 Biological pathways between HIST1H1B, HIST1H2BN and 4 traits.**


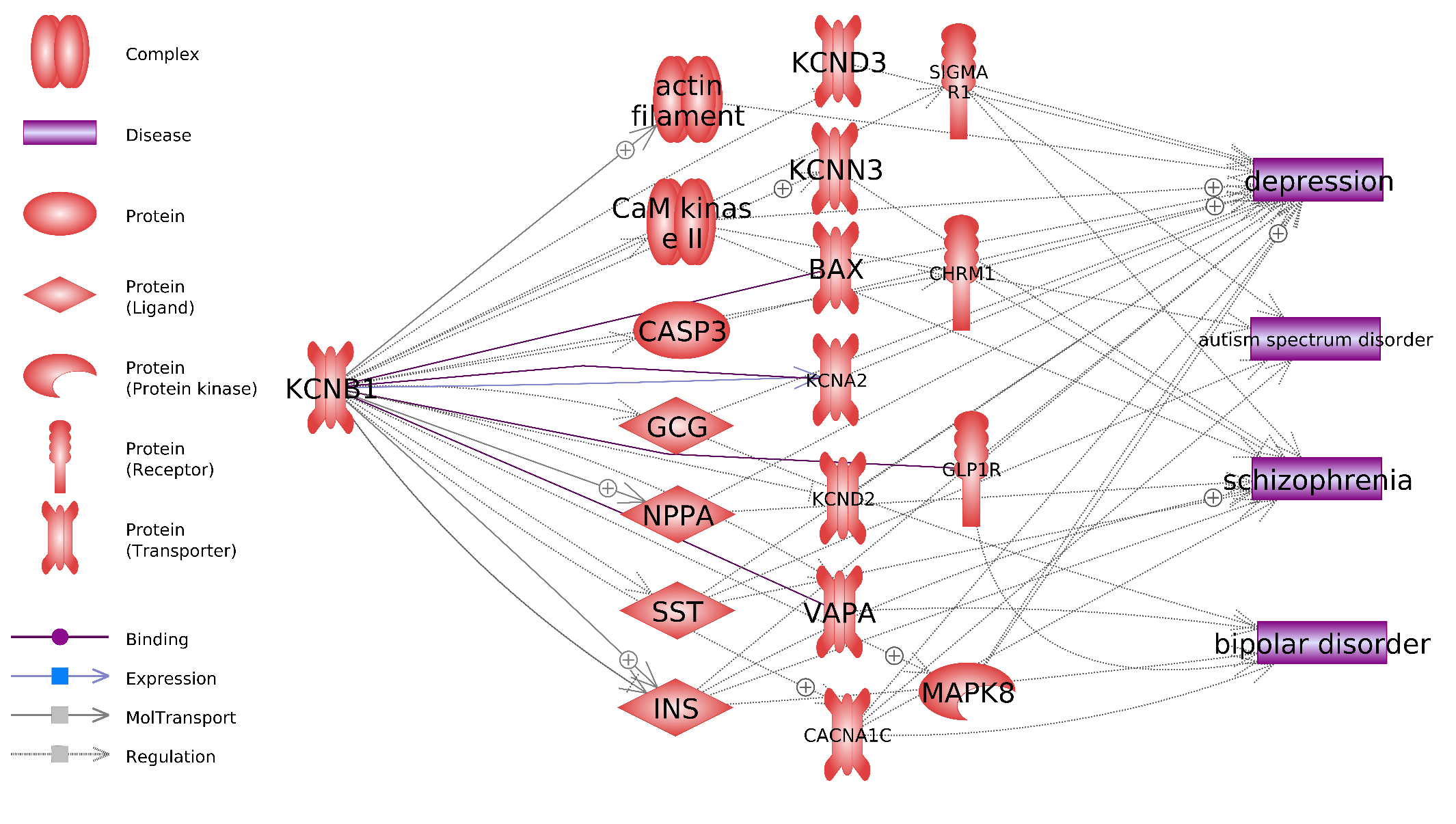


**Figure 10 Biological pathways analysis between KCNB1 and 4 traits**

Supplementary note 1 Information for the data

| Panel | N |
| --- | --- |
| Attention Deficit Hyperactivity Disorder(ADHD) | 55374 |
| Autism Spectrum Disorder(ASD) | 46350 |
| Depression(DEP) | 688809 |
| Bipolar Disorder(BD) | 41653 |
| Schizophrenia (SCZ) | 65967 |

1 Grove, J., et al., *Identification of common genetic risk variants for autism spectrum disorder.* Nat Genet, 2019. **51**(3): p. 431-444

2. Demontis, D., et al., *Discovery of the first genome-wide significant risk loci for attention deficit/hyperactivity disorder.* Nature genetics, 2019. **51**(1): p. 63.

3. Bipolar, D., et al., *Genomic Dissection of Bipolar Disorder and Schizophrenia, Including 28 Subphenotypes.* Cell, 2018. **173**(7): p. 1705-1715 e16.

4. Nagel, M., et al., *Meta-analysis of genome-wide association studies for neuroticism in 449,484 individuals identifies novel genetic loci and pathways.* Nature Genetics, 2018. **50**(7): p. 920

**Supplementary note 2 DEP sample**

It needs to be emphasized that GWAS on a depressive symptoms’ phenotype was performed in PLINK using a linear regression model. Subsequently, meta-analyses were conducted on UK Biobank (UKB) dataset, the 23andMe depression data, and depression data from the Psychiatric Genome Consortium (PGC). In this study, depression, including major depression disorder patients and others, was operationalized by adding up the scores on two continuous items (“Over the past two weeks, how often have you felt down, depressed or hopeless?” and “Over the past two weeks, how often have you had little interest or pleasure in doing things?”; both were evaluated on a four-point Likert scale: from ‘not at all’ to ‘nearly every day’), resulting in a continuous depression score.

**Supplementary note 3 FUMA**

FUMA was mainly used for implementing analysis. FUMA is a comprehensive web-based platform that provides a series of data resources/tools such as, 1000 Genomes projects phase 3, ANNOVAR, MAGMA and the Genotype-Tissue Expression (GTEx), to facilitate functional annotation, gene prioritization and interactive visualization for post-GWAS annotation. FUMA can translate the original GWAS result into testable functional hypothesis. Genetic variants for each dataset were annotated and analyzed using FUMA.

All the parameters we used in FUMA were default.
